# The epiGenomic Efficient Correlator (epiGeEC) tool allows fast comparison of user datasets with thousands of public epigenomic datasets

**DOI:** 10.1093/bioinformatics/bty655

**Published:** 2018-07-24

**Authors:** Jonathan Laperle, Simon Hébert-Deschamps, Joanny Raby, David A de Lima Morais, Michel Barrette, David Bujold, Charlotte Bastin, Marc-Antoine Robert, Jean-François Nadeau, Marie Harel, Alexei Nordell-Markovits, Alain Veilleux, Guillaume Bourque, Pierre-Étienne Jacques

**Affiliations:** 1 Département d’Informatique, Université de Sherbrooke, Sherbrooke, QC, Canada; 2 Département de Biologie, Université de Sherbrooke, Sherbrooke, QC, Canada; 3 Centre de Calcul Scientifique, Faculté des Sciences, Université de Sherbrooke, Sherbrooke, QC, Canada; 4 McGill University and Génome Québec Innovation Center, Montréal, Canada; 5 Department of Human Genetics, McGill University, Montréal, Canada; 6 Centre de Recherche du CHUS, Université de Sherbrooke, Sherbrooke, QC, Canada

## Abstract

**Summary:**

In recent years, major initiatives such as the International Human Epigenome Consortium have generated thousands of high-quality genome-wide datasets for a large variety of assays and cell types. This data can be used as a reference to assess whether the signal from a user-provided dataset corresponds to its expected experiment, as well as to help reveal unexpected biological associations. We have developed the epiGenomic Efficient Correlator (epiGeEC) tool to enable genome-wide comparisons of very large numbers of datasets. A public Galaxy implementation of epiGeEC allows comparison of user datasets with thousands of public datasets in a few minutes.

**Availability and implementation:**

The source code is available at https://bitbucket.org/labjacquespe/epigeec and the Galaxy implementation at http://epigeec.genap.ca.

**Supplementary information:**

Supplementary data are available at *Bioinformatics* online.

## 1 Introduction

Processing files generated by high-throughput sequencing of epigenomic experiments should involve evaluating the quality of both the raw reads and their alignment. Confirming that the signal in a resulting file globally corresponds to the expected experiment is also highly desirable before entering the time-consuming analysis and interpretation steps. However, determining for instance the biological assay and the cell type is virtually impossible by looking at the signal without comparative datasets. This is usually available only to groups producing enough data themselves. Using high-quality reference data generated in a large variety of assays and cell types by consortia such as the International Human Epigenome Consortium (IHEC) (Stunnenberg *et al.*, 2016), it becomes possible to develop tools for the validation of user datasets. The IHEC Data Portal (epigenomesportal.ca/ihec/grid.html) (Bujold *et al.*, 2016) provides such processed datasets, while public databases such as Gene Expression Omnibus (GEO) (Barrett *et al.*, 2013) are giving access to raw epigenomic data.

Comparing large numbers of datasets simultaneously in a reasonable amount of time requires an efficient approach. The epiGenomic Efficient Correlator (epiGeEC) tool was developed specifically to fulfill this need. A few other tools such as bigWigCorrelate (Kent *et al.*, 2010) and DeepTools (Ramírez *et al.*, 2016) have the capability to generate correlation matrices on many datasets, but they need to read each signal file every time a new dataset is to be compared, which greatly affects performance. Instead, epiGeEC uses intermediate files in the high-performance HDF5 format. A user-friendly public Galaxy interface integrating epiGeEC and featuring a large collection of reference datasets enables the efficient comparison of user-provided datasets with thousands of public epigenomic datasets in only a few minutes (http://epigeec.genap.ca/). This public Galaxy framework is provided by the Genetics and genomics Analysis Platform (GenAP) project, thereby leveraging Compute Canada advanced research computing infrastructure.

## 2 Methods

EpiGeEC is designed to efficiently perform pairwise correlations of thousands of epigenomic datasets. It supports many genomic signal file formats (bigWig, WIG and bedGraph), and offers the possibility of computing correlations at various resolutions (from 1 kb to 1 Mb) on pre-defined filtered regions (e.g. whole genome with or without blacklisted regions, only genes, TSS) and using the selected correlation metric (Pearson and Spearman), as well as the annotation and analysis of the generated correlation matrices. Most of the wrapping is coded in Python, while the core functionalities requiring high performance are coded in C++ using the openMP API for parallelization.

Public datasets gathered in the epiGeEC server currently include more than 10 000 processed datasets from IHEC (human hg19 and hg38 and mouse mm10 assemblies), as well as a subset of ∼1000 high-quality ChIP-Seq and chromatin accessibility data from the yeast model organism *Saccaromyces cerevisiae* downloaded from GEO and uniformly processed on the sacCer3 assembly.

## 3 Results

**Fig. 1.**
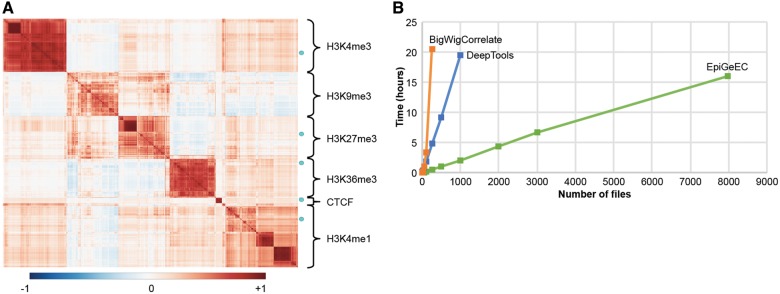
(**A)** Heatmap representation adapted from the PDF report (Supplementary Fig. S2), showing the annotated correlation matrix between 10 user-provided datasets (cyan dots) mapped onto hg19 (accession GSE50893) and 3101 public pre-computed datasets from the IHEC_hg19_2017-10 freeze. This matrix was generated in <10 min. **(B)** Time spent by three tools generating correlation matrices of various sizes, starting directly from the bigWig (no pre-computation)

A typical use case is to compare some signal files to a set of public datasets within minutes. For instance, we uploaded in the epiGeEC-Galaxy history 10 bigWig files from ChIP-Seq experiments conducted on four histone modifications and the CTCF transcription regulator. We then used the Public Dataset Selection tool to select 3101 datasets from the IHEC_hg19_2017-10 freeze, corresponding largely to the same assays (Supplementary Fig. S1). Upon selection, a JSON file containing the metadata from the selected public datasets was uploaded to the history. The Correlation Matrix tool was then used to select the JSON file and the 10 user-provided datasets from the history to compute, in <10 min, a Pearson correlation matrix of 3111 elements at a resolution of 1 kb over the whole genome. The Annotate Matrix tool was then used to generate a report containing a dendrogram, heatmap and pie chart annotations, as well as a multidimensional scaling representation (Supplementary Fig. S2). As shown in Figure 1A, the heatmap representation shows that the five pairs of user datasets (cyan dots) are, as expected, highly similar to histone modifications or CTCF experiments.

As explained in Supplementary Material, one of the reasons for the high performance of epiGeEC is the pre-computation of the signal in intermediate HDF5 files, thereby reducing the time limiting step of reading the signal files. We compared the performances of three tools used to generate correlation matrices of various sizes, starting from the bigWig signal files to the final matrix at a resolution of 1 kb. While the matrices generated with the three tools were highly similar (average Pearson coefficient >0.93, Supplementary Table S1), epiGeEC was ∼10 times faster than DeepTools (version 2.4.3) and 5–40 times faster than bigWigCorrelate, while using more than 10 times less peak memory than the other tools (Fig. 1B and Supplementary Fig. S3). Because of these constraints, on a server containing 48 cores and 256 GB of RAM, the largest correlation matrices we were able to generate contained 250 datasets using bigWigCorrelate, and 1000 datasets using DeepTools. By comparison, it took to epiGeEC ∼16 h and ∼96 GB of RAM to create a complete correlation matrix from ∼8000 raw bigWig files from IHEC_hg19_2017-10 freeze.

In recent years, epiGeEC has been used to pre-calculate static correlation matrices that are incorporated to the IHEC Data Portal, and that have been used to identify problematic datasets as part of a quality control pipeline. EpiGeEC has also proven useful to demonstrate that despite the non-uniform procedures applied by the different consortia, the IHEC datasets are overall highly comparable (Breeze *et al.*, 2016). We plan to regularly update the IHEC data and expand the referenced processed datasets from human and model organisms, as well as to develop a programmatic access to the epiGeEC functionalities.

## Supplementary Material

Supplementary Information
