# Supplementary material for: The epiGenomic Efficient Correlator (epiGeEC) tool allows fast comparison of user datasets with thousands of public epigenomic datasets
Source: Bioinformatics. 2018 Jul 24;35(4):674–6. doi: 10.1093/bioinformatics/bty655 (PMC6378939; doi:10.1093/bioinformatics/bty655)
Supplement: Supplementary Information [file bty655_supplementary_information.pdf]

Classification: Biological Sciences: Genome analysis

The epiGenomic Efficient Correlator (epiGeEC) tool allows fast comparison of user datasets with thousands of public epigenomic datasets

Jonathan Laperle[1,2], Simon Hébert-Deschamps[1], Joanny Raby[2], David Anderson de Lima Morais[3], Michel Barrette[3], David Bujold[4], Charlotte Bastin[2], Marc-Antoine Robert[2], Jean-François Nadeau[2], Marie Harel[2], Alexei Nordell-Markovits[2], Alain Veilleux[3], Guillaume Bourque[5] and Pierre-Étienne Jacques[1,2,3,6,*]

[1]Département d'informatique, [2]Département de biologie, [3]Centre de calcul scientifique, Faculté des sciences, Université de Sherbrooke, Sherbrooke, Québec, Canada; [4]McGill University Génome Québec Innovation Center, [5]Department of Human Genetics, McGill University, Montréal, Québec, Canada; [6]Centre de recherche du CHUS, Université de Sherbrooke, Sherbrooke, Québec, Canada

*To whom correspondence should be addressed.

Supplementary information

Supplementary text

The epiGeEC command line tools

EpiGeEC supports the most popular genomic file formats used to represent signal such as bigWig, WIG and bedGraph. Using the `to_hdf5` utility, a signal file is processed into the efficient HDF5 format by averaging the signal in bins of a given size. Each chromosome is stored as an array in the HDF5 file that also contains pre-computed information to accelerate the following correlations. It is also possible to filter the signal files to select or exclude regions by providing corresponding BED files.

The `correlate` utility is taking a list of HDF5 files to compute pairwise correlations using the Pearson or Spearman metric to create a matrix. Providing this correlation matrix to the `annotate` utility along with a file containing the metadata (in JSON format) produces a hierarchical clustering of the matrix and generates a visual representation as a heatmap and dendrogram, with the possibility to annotate a given (or automatically identified) number of clusters with pie charts. The `evaluate` utility can be used to assess the accuracy of the clustering using the Adjusted Rand Index (ARI) measure (Rand, 1971). The ARI scores are ranging from 0 to 1, with high values indicating that the structure of the clusters fit to the structure of the provided metadata. It is also possible to select subsets of an existing correlation matrix using the `slice` utility. The source code of epiGeEC along with some reference files is available at https://bitbucket.org/labjacquespe/epigeec.

The public epiGeEC Galaxy server

Public datasets gathered in the epiGeEC server currently include more than 10,000 processed datasets from IHEC human hg19 and hg38 assemblies, in addition to the mouse mm10, as well as a subset of ~1000 high quality ChIP-Seq and chromatin accessibility data from the yeast model organism *Saccaromyces cerevisiae*. The yeast data was downloaded from GEO and uniformly processed using Trimmomatic (Bolger *et al.*, 2014), BWA for the mapping onto the sacCer3 assembly (using aln or mem depending on the read length) (Li and Durbin, 2009), BEDTools genomecov to calculate read density (Quinlan and Hall, 2010), and Kent utilities for bigWig conversion (Kent *et al.*, 2010). More than 1500 raw data and metadata from *S. cerevisiae* were downloaded, but we restricted for now to the datasets sequenced with Illumina technology for which there was no ambiguity from the metadata regarding the target, and having >2M mapped reads representing >40% of the reads mapped with a quality >20. The metadata of the human and mouse samples was imported from the IHEC Data Portal

(http://epigenomesportal.ca/ihec/grid.html) and follow IHEC metadata specification available on the GitHub repository (https://github.com/IHEC/ihec-metadata/blob/master/specs/Ihec_metadata_specification.md).

From the Galaxy interface, the "Get Data" menu on the leftmost panel allows users to upload datasets to their history (the welcome page provides more details), while the "epiGeEC Tools" menu gives access to our current four tools. To facilitate the selection of the public datasets among the thousands available, we provide the user-friendly `Public Dataset Selection` interface (epigeec-datasets.udes.genap.ca) linked to GenAP-Galaxy (Fig. S1) (the option to refine a previous selection or import a IHEC Data Portal session ID will soon be available). Upon selection of the desired datasets, a JSON file containing their metadata is downloaded to the user's history. From the `Correlation Matrix` tool interface, it is possible to select such a JSON file of public datasets and/or individual user datasets from the history, to correlate at a given resolution (from 1 kb to 1 Mb) on predefined filtered regions (e.g. whole genome with or without blacklisted regions, only genes, TSS) and using the selected correlation metric (Pearson, Spearman). Complete correlation matrices were pre-computed from all gathered public datasets, while selected user datasets are internally converted to equivalent binned and filtered HDF5 files to be used for pairwise correlations. The refSeq gene annotations were extracted from UCSC on January 2017.

The resulting matrix is output in the history and can be used by the `Annotate Matrix` tool to generate a PDF report containing the dendrogram, heatmap and pie charts (FigS2A), as well as a tabular (TSV) file containing the ordered metadata after clustering. In a case where a user dataset is selected, it will be clearly identified in the heatmap representation and easy to identify in the TSV, allowing to easily determine to which other datasets it is the most similar. A multidimensional scaling (MDS) representation of the correlation matrix can also be generated (FigS2B). The `Evaluate Clustering` tool can then be used to assess how well the structure of the clusters corresponds to the provided metadata for each category of labels. For instance, the ARI scores calculated on the IHEC public datasets generated from core assays clearly indicate that they mainly clustered based on the assay type and sample cell type, rather than on the producing consortium.

The default parameters of the `Correlation Matrix` tool (1 kb bins covering the whole genome with the blacklisted regions excluded (except for hg38 for which no blacklisted regions have been identified)) were determined based on the analysis of various sets of IHEC datasets on which ARI scores were computed. In general the ARI scores for the assays and cell types label categories were similar between 100 bp and 1 kb bins (although slightly better at 1 kb and using 10 times less memory), but were decreasing until 1 Mb (and very bad using bins of 10 Mb). We nonetheless decided to offer in Galaxy the resolutions from 1 kb to 1 Mb in cases where bins >1 kb would be more suitable for some users. Selecting subsets of the genome such as genes or TSS was also giving very interesting ARI scores for the distinction of the assays, but much less for the distinction of the cell type, in particular for some assays (e.g. as expected, it is much harder to obtain good cell type clusters using only TSS bins with the H3K36me3 assay, than using all the bins). Interestingly, selecting the blacklisted regions to compute the correlations was giving quite good ARI results regarding the "publishing_group", indicating that these regions clearly contain signal that is affected by some of the experimental settings specific at each consortium. Similarly, the "average" linkage method of the hierarchical clustering was generally giving slightly better ARI results than the other linkage methods.

Implementation

EpiGeEC tools were developed using python 2.7 with numpy, scipy, pandas and matplotlib for most of the wrapping and post-correlation analyses. The code is also compatible with python 3. The core functionalities, which require high performance, were coded in C++ using the openMP API to parallelize the process, including reading the bigwig files, converting signal files to our intermediate HDF5 format files and finally the correlation itself. The public datasets selection website we created to allow galaxy users access to public datasets was developed mostly in javascript for a fully dynamic user experience with a middleware Django on an Apache2 HTTP server. The communication to Galaxy is setup following the synchronous data deposition procedure described in (Blankenberg *et al.*, 2011) Our bigwig reader, written in C++, is based on the Java implementation of IGV.

The HDF5 files are generated using minimal memory from the data read from the signal files. Arrays of the appropriate length (based on the selected bin size) are first generated and then filled by the program as it reads every entry of the signal file. The correlation has a much higher memory usage as all HDF5 files are loaded into memory as a first step and then correlated in parallel. The performance improvement of using HDF5 format over bigWig is mainly caused by the absence of compression of the HDF5 format and the binning of the data by epiGeEC; to compute the correlations between one user-provided signal file and a thousand pre-computed public signal files, the binned values are directly loaded into memory without other operations. The vast majority of the time spent to read a bigWig file is not bound by the disk access (I/O), but is rather used to decompress the many blocks of a bigWig file. Regarding the binning, the corresponding HDF5 file of a classical ~900 MB bigWig file binned at 1kb is taking ~30 MB, which is ~30 times less to load in memory.

Comparison with other tools

We wanted to compare the performance of epiGeEC with the two other tools that can be used to create correlation matrices on many datasets (Ramírez *et al.*, 2016; Kent *et al.*, 2010). It should be noted that since bigWigCorrelate does not have a resolution parameter, to be as fair as possible, we took ~3000 signal files from IHEC (corresponding to the nine core assays of IHEC generated on 27 different cell-types by 5 consortia) that we filtered to only keep canonical chromosomes (and remove chrom Y), then binned at 1kb and transformed in bigWig files. The binning consists of computing the average signal of non-overlapping windows covering the whole genome, where missing data (positions of the genomes with no signal reported) are replaced by zeros (which particularly impact DeepTools as explained below). The modified pre-binned bigWig files were used for all the benchmarks. The three tools were used to generate correlation matrices of various sizes, starting from the bigWig files to a final matrix of NxN, where N ranges from 10 to 3000, each benchmark was repeated three times and the average was used. The selection of the files for the different subsets was done by ranking the ~3000 files in alphabetical order of their md5sum values, then we selected the first 10, first 25, … up to the first 1000 (which is the equivalent of a random selection). The benchmarks were conducted on a server with access to 48 cores, 256 GB of RAM, and an SSD. To be more specific, both Deeptools and epiGeEC have as a first step the conversion of bigWig files to an internal representation (`multiBigWigSummary` that is creating a NPZ file (Numpy's compressed array format) for Deeptools and `to_hdf5` for epiGeEC); to be able to process 1000 bigWig files with `multiBigWigSummary` at a resolution of 1kb without exceeding the memory limit (256 GB), we had to restrain the number of cores used at this first step to 24, such that for the benchmark we also limited epiGeEC to 24 cores even if it was not required. The second step of both tools (`plotCorrelation` for Deeptools and `correlate` for epiGeEC) was conducted using 48 cores. While it is possible to provide bigWigCorrelate a list of files to generate a correlation matrix, this functionality does not offer parallel processing; we instead decided to parallelize the matrices production by only providing two files at a time in order to offer a fairer comparison and use 48 cores (at the expense of the memory usage). Figure S4 shows that reducing by half the number of cores used by epiGeEC approximately doubles the runtime. As demonstrated by the benchmark results (Fig. 1B and S3), the command line version of epiGeEC outperforms the other tools in the generation of the correlation matrices both for the wall time and the memory usage. And as mentioned in the main text, the Galaxy implementation of epiGeEC is even faster than what we obtain with those benchmarks, since the correlation matrices of public datasets were precomputed, therefore leaving only the correlations between the (usually few) user-provided datasets and the (usually many) selected public ones) to compute.

We also wanted to determine how similar the resulting correlation matrices were between the three tools. We therefore calculated Pearson correlation coefficients between matrices, and obtained coefficients ranging from 0.99 for matrices generated from 10 pre-binned and modified bigWig files, to 0.94 with 1000 files (Tab. S1). It should be noted that using these pre-binned files, DeepTools and bigWigCorrelate return identical correlation matrices, while epiGeEC is generating highly similar but not identical matrices. This can be explained by a difference in the methodology since the default behavior of epiGeEC is to calculate one correlation value per chromosome and to report the weighted average (where weights are function of chromosome length), while the two other tools are concatenating all the genomic values in only one big array. We choose the weighted-chromosomes method as default behavior because it is more robust to outlier values and is less influenced by

strong local correlations. As a result, this method generally produces higher ARI results for biologically relevant labels (e.g. assays), which indicates that the correlation values allow a clearer distinction between datasets sharing different labels. For instances, the ARI score calculated on a correlation matrix generated on the 3101 signal files used in Figure 1A with the weighted-chromosomes method is 0.876 for the clustering of the assays, while it is 0.870 using the concatenated-chromosomes method. One of the biggest differences between the two methodologies is obtained by using the 591 bigWig files from the mRNA-Seq assay (which is one of the assay with the biggest dynamic range and therefore more prone to extreme values): when calculated on the clustering of the cell-types, the ARI score is 0.64 using the weighted-chromosomes method, compared to 0.31 using the concatenated-chromosomes method.

However, it should be noted that the similarity results between the three tools are much lower using the filtered, but non-pre-binned, bigWig files (coefficients ranging from 0.99 to 0.52) (Tab. S2). This particularly affects DeepTools, and seem to be caused by the "missing data" since its default behavior is to remove all the bins with a missing data from all the files, resulting in a reduction of the arrays length as the number of files compared increases. This behavior is probably particularly relevant for some of the many interesting features of DeepTools. The correlation matrices generated by bigWigCorrelate on the filtered files are very similar to the ones generated by epiGeEC (coefficients >0.93), even if it does not use bins. As shown in Table S2, we also implemented in the command-line version of epiGeEC the concatenated-chromosomes method, and as expected the resulting correlation matrices are even more similar to the ones generated by bigWigCorrelate (coefficients >0.96), and identical when using pre-binned bigWig files (with a precision of 5 digits because epiGeEC is using 32 bits floats while bigWigCorrelate is using 64 bits) (not shown).

References

Blankenberg,D. *et al.* (2011) Integrating diverse databases into an unified analysis framework: A Galaxy approach. *Database*, **2011**.

Bolger,A. *et al.* (2014) Trimmomatic: a flexible trimmer for Illumina sequence data. *Bioinformatics*, 1–7.

Kent,W.J. *et al.* (2010) BigWig and BigBed: Enabling browsing of large distributed datasets. *Bioinformatics*, **26**, 2204–2207.

Li,H. and Durbin,R. (2009) Fast and accurate short read alignment with Burrows-Wheeler transform. *Bioinformatics*, **25**, 1754–60.

Quinlan,A.R. and Hall,I.M. (2010) BEDTools: a flexible suite of utilities for comparing genomic features. *Bioinformatics*, **26**, 841–2.

Ramírez,F. *et al.* (2016) deepTools2: a next generation web server for deep-sequencing data analysis. *Nucleic Acids Res.*, **44**, W160–W165.

Rand,M. (1971) Objective Criteria for the Evaluation of Methods Clustering. *J. Am. Stat. Assoc.*, **66**, 846–850.

Supplementary tables

Table S1 - Pearson correlation coefficients calculated on the correlation matrices generated by each tool on different subsets of **pre-binned** bigWig files

**10x10**	**epiGeEC**	**DeepTools**	**bigWigCorrelate**
**epiGeEC**	1.00	0.99	0.99
**DeepTools**	0.99	1.00	1.00
**bigWigCorrelate**	0.99	1.00	1.00

50x50	epiGeEC	DeepTools	bigWigCorrelate
**epiGeEC**	1.00	0.97	0.97
**DeepTools**	0.97	1.00	1.00
**bigWigCorrelate**	0.97	1.00	1.00

100x100	epiGeEC	DeepTools	bigWigCorrelate
**epiGeEC**	1.00	0.96	0.96
**DeepTools**	0.96	1.00	1.00
**bigWigCorrelate**	0.96	1.00	1.00

500x500	epiGeEC	DeepTools	bigWigCorrelate
**epiGeEC**	1.00	0.94	---
**DeepTools**	0.94	1.00	---
**bigWigCorrelate**	---	---	---

1000x1000	epiGeEC	DeepTools	bigWigCorrelate
**epiGeEC**	1.00	0.94	---
**DeepTools**	0.94	1.00	---
**bigWigCorrelate**	---	---	---

Table S2 - Pearson correlation coefficients calculated on the correlation matrices generated by each tool (in addition to the concatenated-chromosomes method explained in the Supplementary text) on different subsets of **non-pre-binned** (but filtered) bigWig files.

10x10	epiGeEC	DeepTools	bigWigCorrelate	*epiGeEC-concat*
**epiGeEC**	1.00	0.92	0.99	*0.99*
**DeepTools**	0.92	1.00	0.93	*0.92*
**bigWigCorrelate**	0.99	0.93	1.00	*0.99*
epiGeEC-concat	*0.99*	*0.92*	*0.99*	*1.00*

50x50	epiGeEC	DeepTools	bigWigCorrelate	*epiGeEC-concat*
**epiGeEC**	1.00	0.84	0.96	*0.97*
**DeepTools**	0.84	1.00	0.82	*0.82*
**bigWigCorrelate**	0.96	0.82	1.00	*0.99*
epiGeEC-concat	*0.97*	*0.82*	*0.99*	*1.00*

100x100	epiGeEC	DeepTools	bigWigCorrelate	*epiGeEC-concat*
**epiGeEC**	1.00	0.80	0.93	*0.96*
**DeepTools**	0.80	1.00	0.75	*0.75*
**bigWigCorrelate**	0.93	0.75	1.00	*0.99*
epiGeEC-concat	*0.96*	*0.75*	*0.99*	*1.00*

**500x500**	**epiGeEC**	**DeepTools**	**bigWigCorrelate**	***epiGeEC-concat***
**epiGeEC**	1.00	0.77	---	*0.94*
**DeepTools**	0.77	1.00	---	*0.72*
**bigWigCorrelate**	---	---	---	---
epiGeEC-concat	*0.94*	*0.72*	---	*1.00*

**1000x1000**	**epiGeEC**	**DeepTools**	**bigWigCorrelate**	***epiGeEC-concat***
**epiGeEC**	1.00	0.52	---	*0.94*
**DeepTools**	0.52	1.00	---	*0.47*
**bigWigCorrelate**	---	---	---	---
epiGeEC-concat	*0.94*	*0.47*	---	*1.00*

Supplementary figures

Figure S1

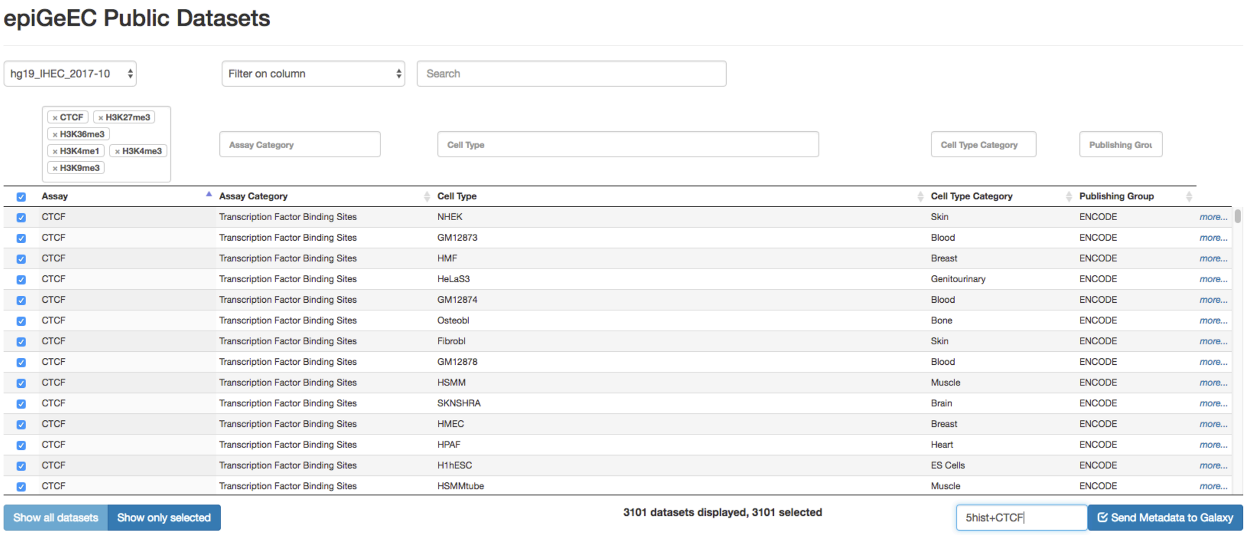

Figure S1. Screenshot of the `Public Dataset Selection` tool showing the selection of the data used in Fig. 1.

Figure S2

A)

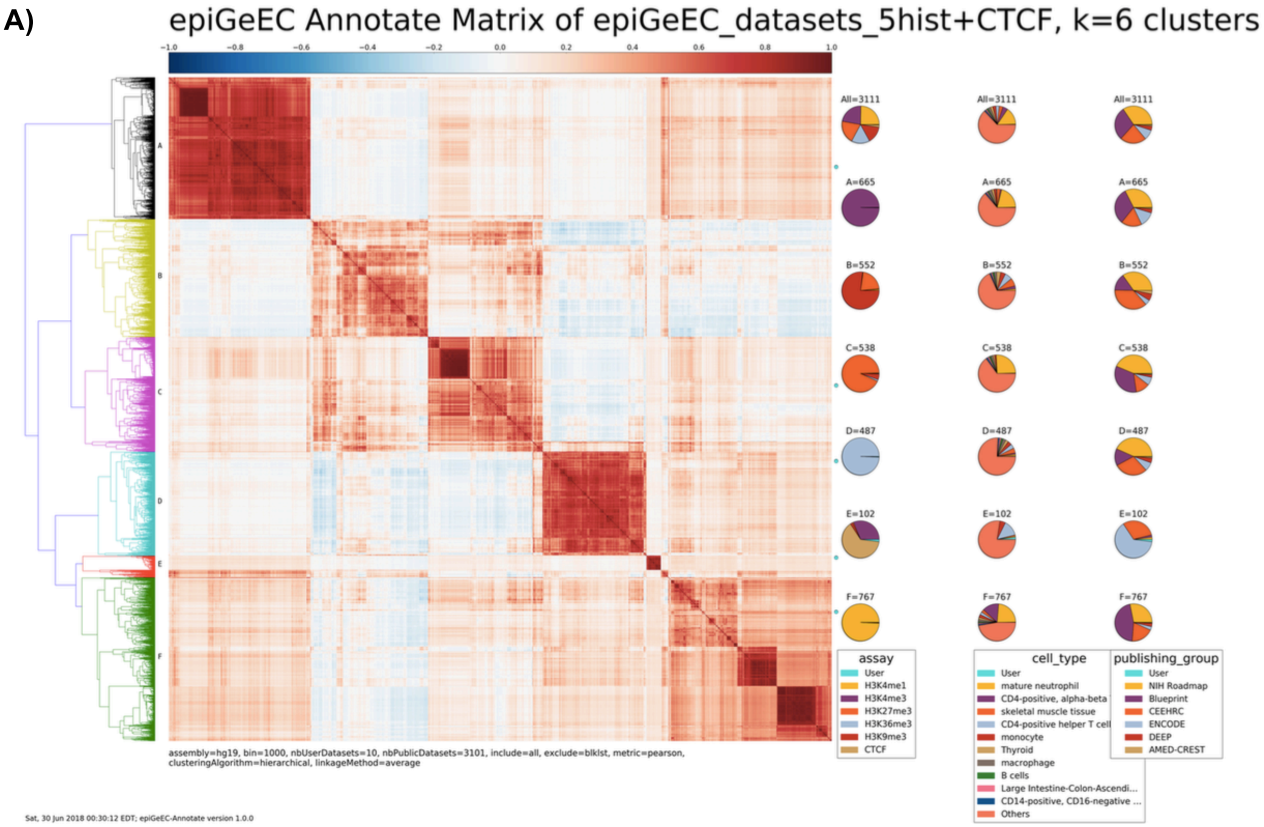

B)

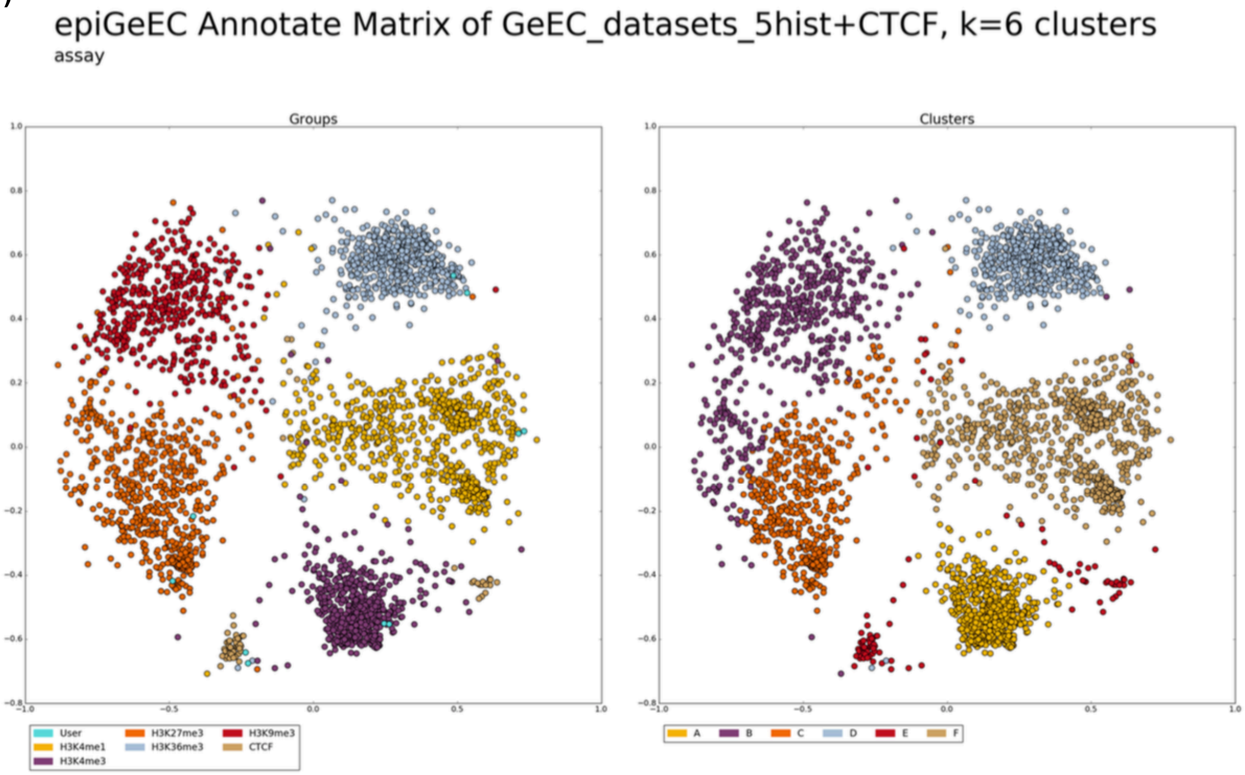

Figure S2. Two of the pages of the PDF report from the `Annotate Matrix` tool showing the user data in cyan. **A)** The dendrogram, heatmap and associated pie charts. **B)** The multidimensional scaling representation.

Figure S3

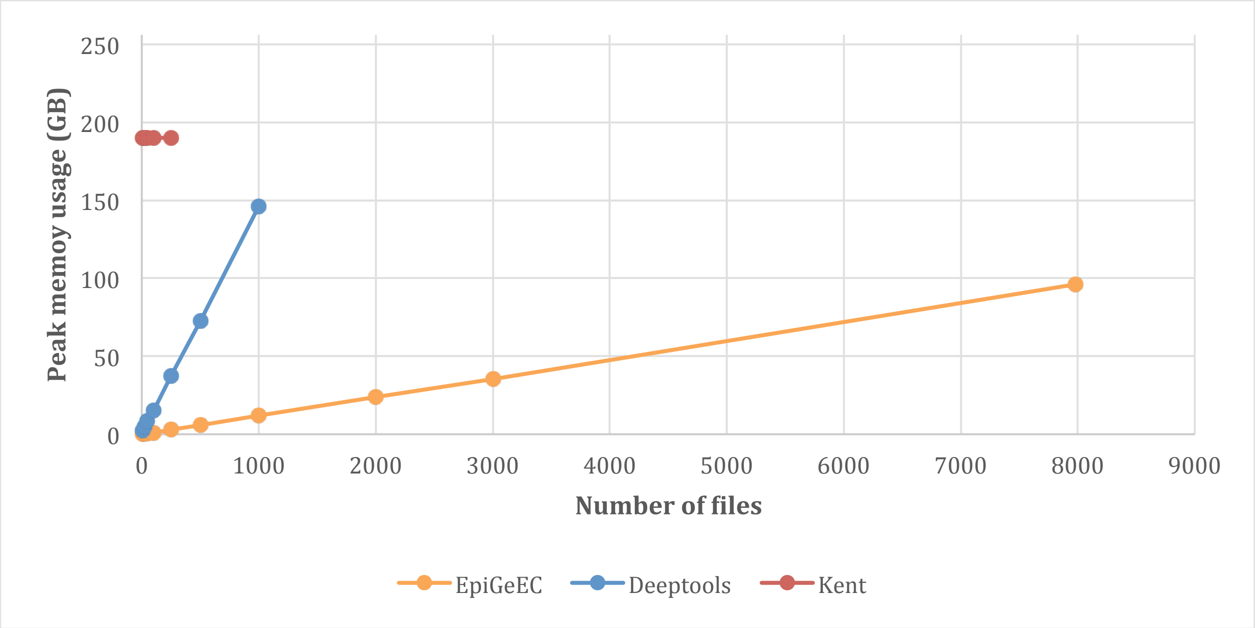

Figure S3. Same comparison as Figure 1B but showing the memory footprint.

Figure S4

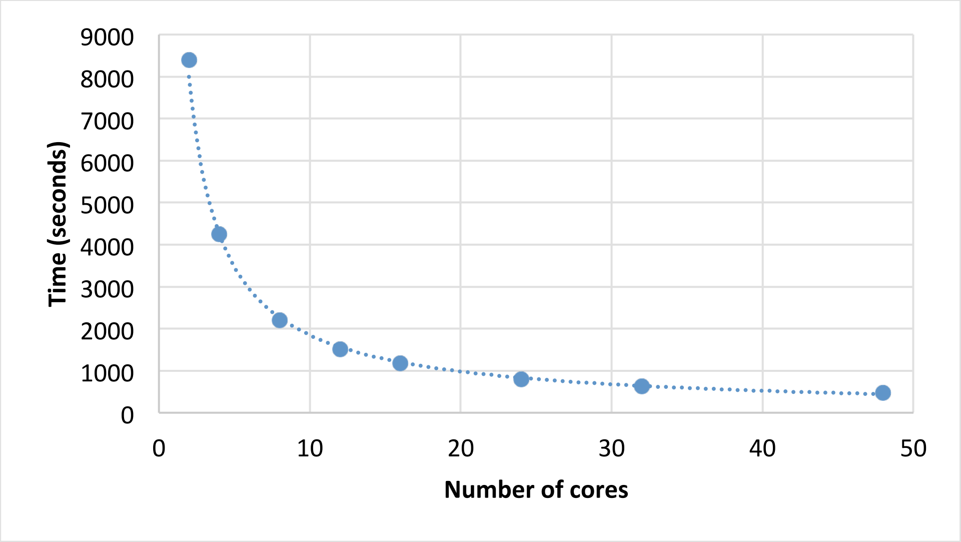

Figure S4. Analysis of the runtime to correlate 100 bigWig files scaled with the number of cores used in parallel.
